# Prognostic factors in aneurysmal subarachnoid hemorrhage with poor initial clinical grade

**DOI:** 10.3389/fneur.2025.1536643

**Published:** 2025-04-02

**Authors:** Diego Culebras, Leire Pedrosa, Alejandra Mosteiro, Laura Llull, Thomaz Topczewski, Luigi Zattera, Laura Díez-Salvatierra, Guillem Dolz, Sergi Amaro, Ramon Torné

**Affiliations:** ^1^Department of Neurosurgery, Hospital Clinic of Barcelona, Barcelona, Spain; ^2^Instituto de Investigaciones Biomédicas August Pi i Sunyer (IDIBAPS), Barcelona, Spain; ^3^Faculty of Medicine, University of Barcelona, Barcelona, Spain; ^4^Comprehensive Stroke Unit, Neurology, Hospital Clinic of Barcelona, Barcelona, Spain; ^5^Neurointensive Care Unit, Department of Anesthesiology and Critical Care, Hospital Clínic de Barcelona, Barcelona, Spain; ^6^Department of Interventional Neuroradiology, Hospital Clínic of Barcelona, Barcelona, Spain

**Keywords:** SAH, WFNS, multimodal monitoring, aneurysm, functional outcome, DCI

## Abstract

**Introduction:**

Aneurysmal subarachnoid hemorrhage (aSAH) is a rare cause of stroke that poses significant morbidity and mortality, as it affects patients around the age of 50 years. While advances in early aneurysm intervention have reduced mortality rates, many patients still experience poor outcomes due to early brain injury (EBI) and delayed cerebral ischemia (DCI). This study aims to explore the characteristics of patients with poor neurological outcomes among patients with poor neurological status at admission, using comprehensive clinical and neuroimaging data.

**Methods:**

We analyzed 377 aSAH patients (WFNS 4–5) admitted between 2013 and 2020, focusing on demographics, clinical assessments, imaging, treatments, and outcomes at discharge and 3 months later.

**Results:**

Among the cohort, which predominantly consisted of females, the mortality rate was 49%. Our findings indicate that older patients had poorer functional outcomes; notably, 59% of patients aged 75 and older had limitations on therapeutic efforts, leading to a 100% mortality rate in that subgroup. There was no difference in outcomes between endovascular and surgical treatments. However, patients undergoing multimodal monitoring had better functional outcomes at discharge. Angiographic vasospasm was found in 31% of patients and was linked to poorer outcomes at discharge (*p* = 0.016). Though DCI did not directly correlate with functional outcomes, it correlated strongly with new cerebral infarcts (90% incidence).

**Conclusion:**

The prognosis of patients with aSAH and poor neurological status on admission is generally poor. Multimodal monitoring and tailored treatment appear to be beneficial in achieving favorable results in these patients. Despite the initial severity, up to 20% of patients achieve a good functional result on discharge and up to 35% do so at 3 months. These should be considered in the initial prognostic assessment with the families of these patients.

## 1 Introduction

Subarachnoid hemorrhage caused by a ruptured aneurysm (aSAH), has a relatively low incidence of 6–10 cases per 100,000 people per year. However, it remains a major cause of stroke-related years of potential life loss, given its high morbidity and mortality rates, particularly among the middle-aged population (1, 2). Over the last decade, there has been a notable decrease in fatal rates associated with aSAH, primarily attributed to successful aneurysm treatment, which significantly reduces the risk of re-bleeding (3–6).

Unfortunately, a significant percentage of patients who receive early treatment and intensive care still do not achieve favorable neurological outcomes. Several causes are attributable to this fatal outcome: among them, there is early brain injury (EBI), which is the damage produced in the first 72 h after the event, as well as delayed cerebral ischemia (DCI), both related to the severity of initial damage (7–9). During this period, several biomarkers have been associated with poor functional prognosis in the short and medium term (10–13).

Many life-saving measures are taken during this early period. Yet, current grading scales are mainly based on clinical and basic radiological data, especially at the initial stage. The clinical status at hospital admission appears to have the greatest impact on the prognosis of these patients, with around 20–30% of all admitted aSAH patients meeting the criteria of high grade (World Federation of Neurological Surgeons (WFNS) 4 or 5).

Under these circumstances, the treatment of patients with poor initial neurological status is often questioned, considering that the reduction in mortality is at the expense of a worse functional prognosis. However, recent studies show that good functional results could be achieved in up to 30% of these patients if optimally treated (14–16). The identification of this subgroup could require multimodal monitoring and a comprehensive assessment approach, including key data obtained with intracranial pressure (ICP), tissue oxygen pressure (PtO_2_), and microdialysis (MD) (17–20).

The objective of this study is to identify which clinical, radiological and systemic factors are associated with good or poor prognosis. For this purpose, we evaluated potential predictive factors using an integrative approach that includes neuroimaging tools [computed tomography angiography (CT angiography), brain magnetic resonance imaging (MRI)] and multimodal neuromonitoring.

## 2 Methods

### 2.1 Patients

This is a retrospective observational study of prospectively collected data. Inclusion criteria: HSAs with poor initial neurological status (WFNS 4 or 5) with ≤24 h from the onset of neurological symptoms at our center. Patients with good initial clinical status (WFNS <4), those without angiographical evidence of aneurysmal origin, and those who were previously functionally dependent (defined as a modified Rankin Scale (mRS) score > 3) were excluded from the analysis. All patients were subsequently monitored according to the detailed protocol. This study was approved by the ethics committee of our center (HCB/2018/0390).

### 2.2 Variables

The following variables were collected: age, sex, risk factors (smoking, diabetes, dyslipidemia, hypertension, and alcoholism), WFNS and Glasgow Coma Scale (GCS) at hospital admission, limitation of therapeutic effort (LTE), modified Fisher scale (mFisher) on initial CT, hydrocephalus at hospital admission, presence of intraparenchymal hematoma (IPH), type of treatment (surgical or endovascular), use of invasive neuromonitoring and type (ICP, PtO_2_ and MD), presence of new cerebral infarction or delayed cerebral ischemia (DCI), appearance of angiographic vasospasm (aVE) (diagnosed by CT angiography, transcranial Doppler ultrasound, or arteriography). Functional outcomes were registered at discharge and 3 months follow-up, using mRS.

We defined delayed cerebral ischemia (DCI) as the onset of focal neurological deficits—including hemiparesis, aphasia, apraxia, hemianopia, or a reduction of at least 2 points on the GCS that persists for at least 1 hour. This deterioration must not be observed immediately following the occlusion of the aneurysm and should not be attributable to other factors, as confirmed by a thorough clinical evaluation, imaging studies (CT or MRI of the brain), and relevant laboratory tests (5).

Cerebral infarction was defined as the presence of an irreversible ischemic injury observed in brain CT or MRI within 6 weeks following the SAH. This injury could not be attributed to treatment or EBI, thus should not be present in the CT or MRI performed between 24 to 48 h after the aneurysm was treated (5).

### 2.3 Initial clinical management

The diagnosis of aSAH was based on non-contrast CT and angioCT scans, performed at our center or the referring center. Immediately upon arrival at our center, the clinical status of the patient was assessed using the GCS and WFNS scales. All patients with a WFNS score of 4 or 5 were admitted to the intensive care unit (ICU). All patients underwent early angiography (24-48 h after the event), except for those in whom the LTE was decided. During admission, prophylactic treatment with oral or intravenous nimodipine was started and maintained for at least 14 days. Intensive hemodynamic management was performed in the ICU to maintain normotension and euvolemia. The definitive treatment of the aneurysm (endovascular or surgical) was agreed upon by the neuroradiology, neurosurgery and neurology teams. An external ventricular drain (EVD) was placed in those patients with symptomatic acute hydrocephalus.

### 2.4 Multimodal monitoring in severe aSAH

**Figure 1:**
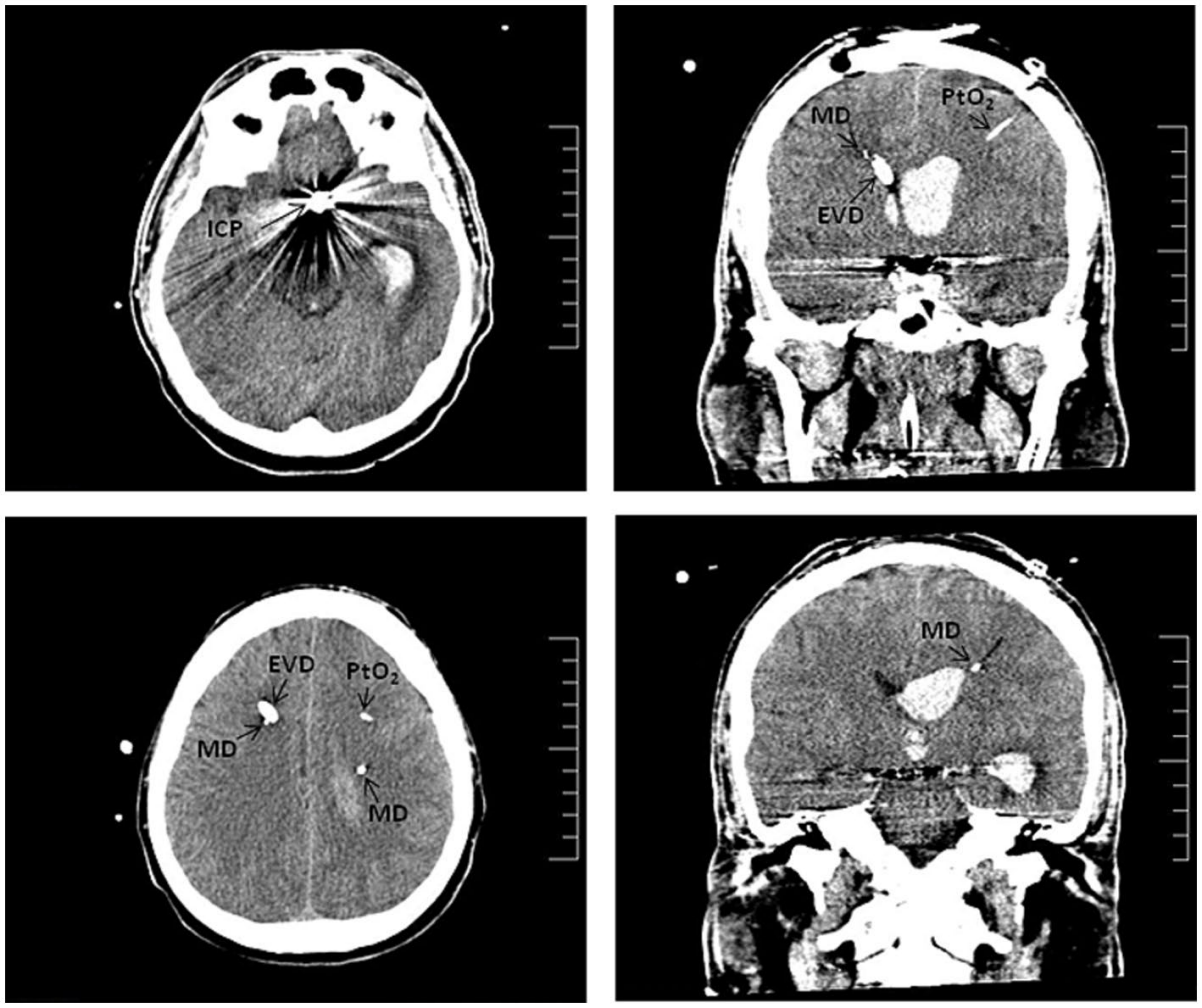
Computed tomography scan of aSAH-patient with poor initial clinical status. SAH due to rupture of an aneurysm in the left ACom. Axial (left panels) and coronal (right panels) sections show the ICP sensors, the catheters for bilateral microdialysis monitoring (MD), the external ventricular drain (EVD) and the PtO2 sensor.

A new neurocritical care protocol was implemented in our center in 2018. This contemplates the use of invasive multimodal neuromonitoring in high-grade aSAH patients expected to require deep sedation with assisted ventilation for at least 96 h, and no less than 24 h. Monitoring includes intraparenchymal/intraventricular ICP sensor, PtO2 sensor, and bilateral MD catheters, all intended to predict ischemic events and optimize the systemic management of these patients (17, 21) (Figure 1).

We considered ICP values below 20–22 mmHg as normal, PtO2 values between 20 and 30 mmHg, and defined ischemic events based on a lactate-to-pyruvate ratio (LPR) greater than 40, along with a glucose concentration below 0.7 mmol/L in the case of microdialysis. These thresholds are critical for predicting ischemic events and optimizing both systemic management and, when necessary, surgical interventions for our patients. The therapeutic measures adopted based on the data obtained from the multimodal neuromonitoring were individualized for each case. These measures ranged from optimizing medical treatment to therapeutic angiographies, and in some cases, surgical interventions as needed.

Bilateral MD catheters were implanted immediately following surgical or endovascular treatment of the aneurysm. The catheters were placed in the operating room using a mini-drill at Köcher point. A CT scan was done in the first 24 h to verify the position of the catheters within the subcortical matter of the watershed territory between the middle cerebral artery (MCA) and anterior cerebral artery (ACA). An EVD was implanted in case of acute hydrocephalus, while a PtO2/ICP was selected if an EVD was not required.

### 2.5 Clinical follow-up

The functional status was evaluated in an ad-hoc follow-up scheduled in the first 2 weeks after hospital discharge and again after 3 months. The score on the mRS scale was registered by the physician or a trained nurse, with a score from 0 to 3 considered a good functional outcome.

### 2.6 Statistics

The continuous numerical variables were summarized using the mean and standard deviations (SD) and were compared using either the Student’s t-test or the Mann–Whitney test, depending on whether they followed a normal distribution or not, respectively. The distribution of the continuous variables was assessed with the Shapiro test. Categorical variables were compared using the Chi-square test. A univariate analysis was performed to evaluate the clinical and radiological variables associated with the mRS at discharge and at 3 months follow-up. For all tests, the significance level was set at a *p*-value<0.05 (two-tailed). All analyses were performed using IBM-SPSS V26.0 (SPSS Inc., Chicago, IL, USA) and R Commander v.4.1.3 (Vienna, Austria).

## 3 Results

### 3.1 Clinic and demographic characteristics

**Figure 2:**
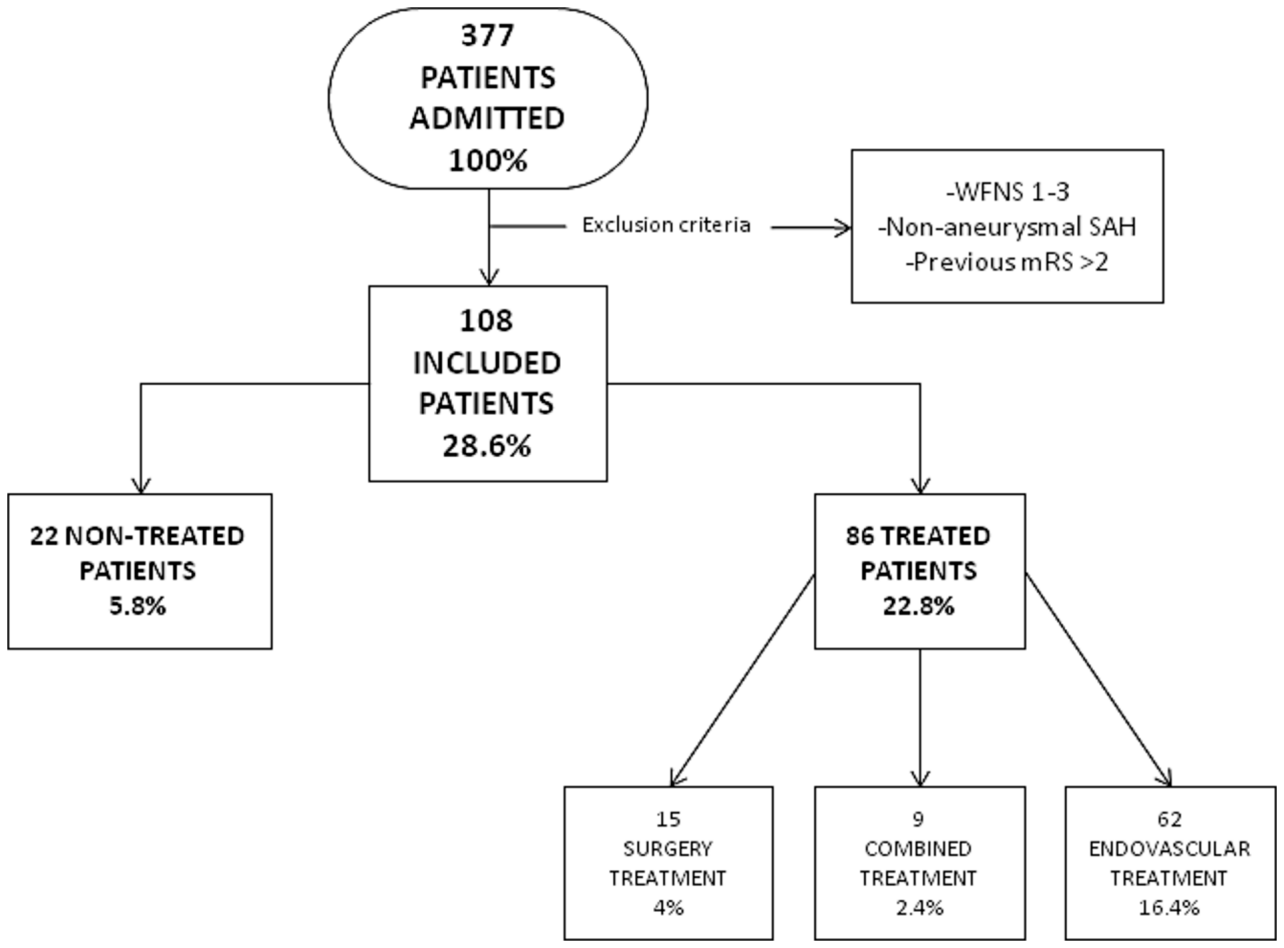
Flow chart of patients admitted with SAH to our center.

Between 2013 and 2020, 377 patients were admitted to our tertiary center with aSAH. Among this cohort, 108 patients had severe aSAH at the time of admission according to the WFNS grades 4–5 and were selected for this study (Figure 2).

Table 1 summarizes the demographic, clinical and angiographic variables of the included patients. The cohort consisted predominantly of females (71/108; 66%), with a mean age of 60 years (SD: 13). The majority were Caucasian (104/108; 96%). Notably, 44% (48/108) of patients had a history of arterial hypertension, and 35% (38/108) were smokers. Most patients had a mFisher score of 4 (102/108; 94%) at admission. The aneurysm locations were primarily in the anterior communicating artery (ACom), accounting for 30% of cases, followed by the left posterior cerebral artery (PCom) at 10% (Table 1).

**Table 1:** Clinical and radiological features of the sample.

	Total	mRS at discharge			mRS at 3 months		
	Total (*n* = 108)	mRS 0–3 (*n* = 23)	mRS 4–6 (*n* = 85)	*p*-value	mRS 0–3 (*n* = 35)	mRS 4–6 (*n* = 73)	*p*-value
Median age (SD)	60.05 (13.04)	54.70 (9.30)	61.49 (13.57)	**<0.0001**	54.70 (9.30)	61.49 (13.57)	**<0.0001**
Age (≥75)	20 (18.5%)	1 (4.3%)	19 (22.3%)	**0.049**	2 (5.7%)	18 (24.7%)	**0.018**
Sex (female)	71 (65.7%)	7 (30.4%)	30 (35.3%)	0.663	25 (28.6%)	46 (37.0%)	0.388
Race				0.466			0.537
Caucasian	104 (96.3%)	22 (95.7%)	82 (96.5%)		34 (97.1%)	70 (95.9%)	
Hispanic	2 (1.9%)	1 (4.3%)	1 (1.2%)		1 (2.9%)	1 (1.4%)	
Other	2 (1.9%)	0	2 (2.3%)		0 (0.0%)	2 (2.7%)	
Smoking	38 (35.2%)	11 (47.8%)	27 (31.8%)	0.152	15 (42.9%)	23 (31.5%)	0.248
Alcohol	8 (7.4%)	2 (8,7%)	6 (7.1%)	0.79	3 (8.6%)	5 (6.8%)	0.749
Hypertension	48 (44.4%)	8 (34.8%)	40 (47.1%)	0.293	15 (42.9%)	33 (45.2%)	0.818
Diabetes	6 (5.6%)	1 (4.3%)	5 (5.9%)	0.776	1 (2.9%)	5 (6.8%)	0.397
Dyslipidemia	29 (26.9%)	8 (34.8%)	21 (24.7%)	0.33	10 (28.6%)	19 (26.0%)	0.78
mFisher				0.869			0.737
2	1 (0.9%)	0	1 (1.2%)		0 (0.0%)	1 (1.4%)	
3	5 (4.6%)	1 (4.3%)	4 (4.7%)		2 (5.7%)	3 (4.1%)	
4	102 (94.4%)	22 (95.7%)	80 (94.1%)		33 (94.3%)	69 (94.5%)	
Aneurysm location				0.756			0.333
ACom	32 (29.6%)	9 (39.1%)	23 (27.1%)		12 (34.3%)	20 (27.4%)	
Left ACA	3 (2.8%)	1 (4.3%)	2 (2.3%)		2 (5.7%)	1 (1.4%)	
Right ACA	1 (0.9%)	0	1 (1.2%)		0 (0.0%)	1 (1.4%)	
Left MCA	10 (9.3%)	2 (8,7%)	8 (9,4%)		3 (8.6%)	7 (9.6%)	
Right MCA	9 (8.3%)	1 (4.3%)	8 (9,4%)		3 (8.6%)	6 (8.2%)	
Left ICA	10 (9.3%)	1 (4.3%)	9 (10.6%)		1 (2.9%)	9 (12.3%)	
Right ICA	6 (5.6%)	1 (4.3%)	5 (5.9%)		2 (5.7%)	4 (5.5%)	
BA	10 (9.3%)	1 (4.3%)	9 (10.6%)		1 (2.9%)	9 (12.3%)	
Left VA	1 (0.9%)	0	1 (1.2%)		0 (0.0%)	1 (1.4%)	
Left PCom	11 (10.2%)	5 (21.7%)	6 (7.1%)		6 (17.1%)	5 (6.8%)	
Right PCom	7 (6.5%)	1 (4.3%)	6 (7.1%)		3 (8.6%)	4 (5.5%)	
PICA	7 (6.5%)	1 (4.3%)	6 (7.1%)		1 (2.9%)	6 (8.2%)	
AICA	1 (0.9%)	0	1 (1.2%)		1 (2.9%)	0 (0.0%)	
WFNS initial				0.275			0.065
4	28 (25.9%)	8 (34.8%)	20 (23.5%)		13 (37.1%)	15 (20.5%)	
5	80 (74.1%)	15 (65.2%)	65 (76.8%)		22 (62.9%)	58 (79.5%)	
GCS initial							0.137
3	49 (45.4%)	9 (39.1%)	40 (47.1%)		12 (34.3%)	37 (50.7%)	
4	11 (10.2%)	1 (4.3%)	10 (11.8%)		2 (5.7%)	9 (12.3%)	
5	6 (5.6%)	2 (8,7%)	4 (4.7%)		2 (5.7%)	4 (5.5%)	
6	7 (6.5%)	1 (4.3%)	6 (7.1%)		2 (5.7%)	5 (6.8%)	
7	8 (7.4%)	1 (4.3%)	7 (8.2%)		1 (2.9%)	7 (9.6%)	
8	8 (7.4%)	1 (4.3%)	7 (8.2%)		4 (11.4%)	4 (5.5%)	
9	5 (4.6%)	1 (4.3%)	4 (4.7%)		3 (8.6%)	2 (2.7%)	
10	3 (2.8%)	1 (4.3%)	2 (2.3%)		2 (5.7%)	1 (1.4%)	
11	5 (4.6%)	2 (8,7%)	3 (3.5%)		3 (8.6%)	2 (2.7%)	
12	4 (3.7%)	2 (8,7%)	2 (2.3%)		2 (5.7%)	2 (2.7%)	
14	2 (1.9%)	2 (8,7%)	0		2 (5.7%)	0 (0.0%)	
LTE	17 (15.7%)	0	17 (20%)	**0.019**	0 (0.0%)	17 (23.3%)	**0.002**
Treatment	86 (79.6%)	23 (100%)	63 (74.1%)	**0.000**	35 (100.0%)	51 (69.9%)	**0.000**
Endovascular	62 (57.4%)	17 (73.9%)	45 (52,9%)	0.755	23 (65.7%)	39 (53.4%)	0.5
Open Surgery	15 (13.9%)	3 (13%)	12 (14.1%)		7 (20%)	8 (10.9%)	
Combined	9 (8.3%)	3 (13%)	6 (7.1%)		5 (14.3%)	4 (5.4%)	
ICP monitoring	86 (79.6%)	21 (91.3%)	65 (76.8%)	0.117	33 (94.3%)	53 (72.6%)	**0.009**
Microdialysis	19 (17.6%)	9 (39.1%)	10 (11.8%)	**0.002**	10 (28.6%)	9 (12.3%)	**0.038**
PtO_2_	22 (20.4%)	7 (30.4%)	15 (17.6%)	0.177	11 (31.4%)	11 (15.1%)	**0.048**
Hydrocephalus	78 (72.2%)	16 (69.6%)	62 (72.9%)	0.748	25 (71.4%)	53 (72.6%)	0.899
ICH	42 (38.9%)	5 (21.7%)	37 (43.5%)	0.057	13 (37.1%)	29 (39.7%)	0.797
Angiographic VE	34 (31.5%)	12 (52.2%)	22 (25.9%)	**0.016**	18 (51.4%)	16 (21.9%)	**0.002**
New cerebral infarction	52 (48.1%)	9 (39.1%)	43 (50.6%)	0.329	13 (37.1%)	39 (53.4%)	0.113
DCI	24 (22.2%)	6 (26.1%)	18 (21.8%)	0.615	8 (22.9%)	16 (17.8%)	0.535

Data are given globally for all SAH patients in the study and dichotomised according to mRS at discharge and at 3 months. Bold numbers correspond to statistical significant differences (*p*-value<0.05).

The overall mortality rate in this cohort was 49% (53/108). Among the deceased patients, most aneurysms were located in the ACom (14/51; 25%) and the left internal carotid artery (ICA) (9/51; 16%).

### 3.2 Age and limitation of therapeutic effort

Patients with good functional outcomes at discharge were younger [mean age 55 years (SD: 9.3)] than patients with poor functional outcomes [mean age 61 years (SD: 13.6)] (*p* = 0.044). Similarly, at 3 months follow-up, the mean age of patients with better prognosis was 55 years (SD: 9.3) compared to 61 years (SD: 13.6) for those with a poor functional outcome (*p* < 0.001).

In our cohort, LTE was decided for 17 patients (17/108; 15%). Notably, 59% of the LTE patients were aged 75 years or older (*p* < 0.001). Thus, age over 75 years (20/108; 18%) was a determining factor for the decision of LTE. Among patients aged 75 and older, LTE was documented in 10 patients (10/20; 50%) while it was recorded in only 7 patients (7/88; 8%) younger than 75 years (*p* < 0.001) (Table 2).

**Table 2:** Clinical data of patients according to the limitation of therapy effort (LTE).

	Total (*n* = 108)	Non-LTE (*n* = 91)	LTE (*n* = 17)	*p*-value
Mean age (SD)	60.05 (13.04)	58.25 (11.91)	69.65 (14.93)	**<0.0001**
Age (> or = 75)	20 (18.5%)	10 (11.0%)	10 (58.8%)	**<0.0001**
Sex (female)	71 (65.7%)	59 (64.8%)	12 (70.6%)	0.646
ICH	42 (38.9%)	32 (35.2%)	10 (58.8%)	0.066
Mortality	52 (48.1%)	35 (38.5%)	17 (100.0%)	**0.000**

Bold numbers correspond to statistical significant differences (*p*-value<0.05).

The most frequent location of the aneurysm in patients with LTE was the basilar artery (BA) (5/17; 29%), followed by the ACom (3/17; 18%) and the right MCA (3/17; 18%). 10 out of these 17 patients (59%) had an associated parenchymal hematoma (*p* = 0.066). All patients with LTE decision (17/17; 100%) eventually died.

### 3.3 Initial clinical status and functional prognosis

Regarding the initial clinical status, 32% of patients (35/108) had a WFNS score of 4, and 68% (73/108) had a WFNS score of 5. At discharge, 8 patients with WFNS grade 4 (8/28; 28%) had a good prognosis compared to 15 patients (15/80; 19%) with grade 5 (*p* = 0.275). At 3 months, 13 patients with WFNS grade 4 (13/28; 46%) had a good clinical evolution compared to 22 patients (22/80; 27%) with grade 5 (*p* = 0.065). Regarding the GCS score at admission, most patients were admitted with a GCS score of 3 (49/108; 45%). Patients with good outcomes had a mean GCS score of 6.7 (SD: 3.7), while those with poor outcomes had a lower mean GCS score of 4.8 (SD: 2.5), with a statistically significant difference (*p* = 0.009).

A total of 78 (78/108; 72%) patients presented hydrocephalus requiring the placement of an EVD, without correlation with functional outcome at discharge (*p* = 0.748) or at 3 months follow-up (*p* = 0.899). Out of the 108 patients studied, we observed a notable re-bleeding rate of 19.4% (21/108) prior to aneurysm securement. 15 of them in patients who ended up having a poor prognosis (15/85; 17.6%); compared to 6 (6/23; 26%) of those who ultimately had a better functional prognosis (*p* = 0.54). There were no significant differences in the prognosis related to cardiovascular risk factors, such as arterial hypertension, diabetes, dyslipidemia, alcoholism, smoking, or antiplatelet or anticoagulant drugs (Table 1).

**Figure 3:**
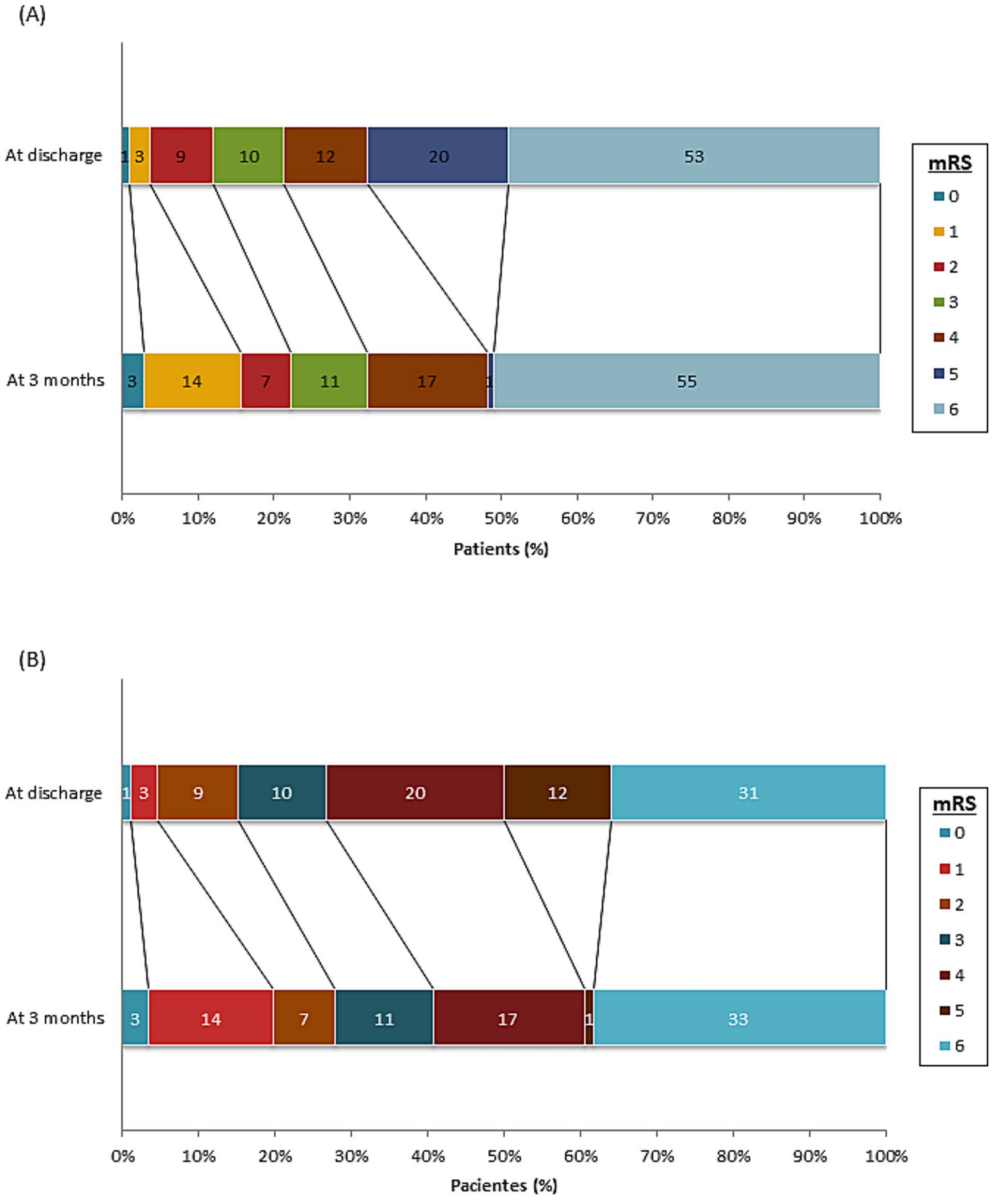
mRS, cumulative, at discharge and at 3 months of all patients included in the study **(A)** and only the treated patients **(B)**.

In total, 23 patients (23/108; 21%) had a good functional outcome at discharge (mRS: 0–3) and 35 (35/108; 32%) at 3 months (Figure 3). In all of them, the cause of the bleeding had been treated.

### 3.4 Aneurysm treatment

Of the 108 patients included in this study, 86 (86/108; 80%) received aneurysm exclusion treatment. 72% (62/86) were treated endovascularly, 17% (15/86) with open surgery, and 10% (9/86) with combined treatment (Figure 2). There were no differences in mortality between the different treatment groups (*p* = 0.08).

Thirty of the treated patients (30/86; 35%) ultimately died. Of these, 26 received endovascular treatment (26/30; 51%), 2 open surgical treatment (2/30; 4%), and 2 combined treatment (2/30; 4%). No significant differences were observed in the type of treatment and functional outcome at discharge (*p* = 0.755) or at 3 months (*p* = 0.5).

The overall mortality of patients who underwent aneurysm exclusion treatment was 35% (30/86) versus 100% (22/22) of those who did not undergo treatment (*p* < 0.001) (Table 3).

**Table 3:** Clinical and radiological features of the treated patients.

Treated patients	Total (*n* = 86)	mRS 0–3 (*n* = 35)	mRS 4–6 (*n* = 51)	*p*-value
Mean age (SD)	58.03 (11.60)	54.09 (10.26)	60.75 (11.77)	**0.000**
Age (> or = 75)	9 (10.5%)	2 (5.7%)	7 (13.7%)	0.233
Sex (female)	55 (64.0%)	25 (71.4%)	30 (58.8%)	0.232
Race				0.684
Caucasian	83 (96.5%)	34 (97.1%)	49 (96.1%)	
Hispanic	2 (2.3%)	1 (2.9%)	1 (2.0%)	
Other	1 (1.2%)	0 (0.0%)	1 (2.0%)	
Smoking	30 (34.9%)	15 (42.9%)	15 (29.4%)	0.199
Alcohol	6 (7.0%)	3 (8.6%)	3 (5.9%)	0.631
Hypertension	35 (40.7%)	15 (42.9%)	20 (39.2%)	0.736
Diabetes	5 (5.8%)	1 (2.9%)	4 (7.8%)	0.332
Dyslipidemia	27 (31.4%)	10 (28.6%)	17 (33.3%)	0.64
mFisher	0	0	0	0.706
2	1 (1.2%)	0 (0.0%)	1 (2.0%)	
3	5 (5.8%)	2 (5.7%)	3 (5.9%)	
4	80 (93.0%)	33 (94.3%)	47 (92.2%)	
Aneurysm location				0.566
ACom	27 (31.4%)	12 (34.3%)	15 (29.4%)	
Left ACA	3 (3.5%)	2 (5.7%)	1 (2.0%)	
Right ACA	0 (0.0%)	0 (0.0%)	0 (0.0%)	
Left MCA	9 (10.5%)	3 (8.6%)	6 (11.8%)	
Right MCA	6 (7.0%)	3 (8.6%)	3 (5.9%)	
Left ICA	6 (7.0%)	1 (2.9%)	5 (9.8%)	
Right ICA	4 (4.7%)	2 (5.7%)	2 (3.9%)	
BA	5 (5.8%)	1 (2.9%)	4 (7.8%)	
Left AV	1 (1.2%)	0 (0.0%)	1 (2.0%)	
Left ACoP	10 (11.6%)	6 (17.1%)	4 (7.8%)	
Right ACoP	7 (8.1%)	3 (8.6%)	4 (7.8%)	
PICA	7 (8.1%)	1 (2.9%)	6 (11.8%)	
AICA	1 (1.2%)	1 (2.9%)	0 (0.0%)	
WFNS on admission				0.248
4	26 (30.2%)	13 (37.1%)	13 (25.5%)	
5	60 (69.8%)	22 (62.9%)	38 (74.5%)	
GCS on admission				0.292
3	35 (40.7%)	12 (34.3%)	23 (45.1%)	
4	10 (11.6%)	2 (5.7%)	8 (15.7%)	
5	5 (5.8%)	2 (5.7%)	3 (5.9%)	
6	5 (5.8%)	2 (5.7%)	3 (5.9%)	
7	6 (7.0%)	1 (2.9%)	5 (9.8%)	
8	8 (9.3%)	4 (11.4%)	4 (7.8%)	
9	4 (4.7%)	3 (8.6%)	1 (2.0%)	
10	3 (3.5%)	2 (5.7%)	1 (2.0%)	
11	4 (4.7%)	3 (8.6%)	1 (2.0%)	
12	4 (4.7%)	2 (5.7%)	2 (3.9%)	
14	2 (2.3%)	2 (5.7%)	0 (0.0%)	
LTE on admission	0 (0.0%)	0 (0.0%)	0 (0.0%)	
ICP monitoring	80 (93.0%)	33 (94.3%)	47 (92.2%)	0.703
Microdialysis	18 (20.9%)	10 (28.6%)	8 (15.7%)	0.149
PtO2	21 (24.4%)	11 (31.4%)	10 (19.6%)	0.210
Hydrocephalus at admission	65 (75.6%)	25 (71.4%)	40 (78.4%)	0.458
ICH	31 (36.0%)	13 (37.1%)	18 (35.3%)	0.861
Angiographic VE	33 (38.4%)	18 (51.4%)	15 (29.4%)	**0.039**
New cerebral infarction	48 (55.8%)	13 (37.1%)	35 (68.6%)	**0.004**
DCI	20 (23.3%)	8 (22.9%)	12 (23.5%)	0.942

Data are given globally for treated patients and dichotomised according to mRS at 3 months. Bold numbers correspond to statistical significant differences (*p*-value<0.05).

### 3.5 Multimodal monitoring

Following the change in our institutional protocol, multimodal monitoring (ICP + PtiO_2_ + cerebral MD) was performed in 20 (20/108; 18%) patients. The combination of ICP + PtiO_2_ was used in 6 (6/108, 6%), while isolated ICP monitoring was performed in 60 patients (60/108, 56%). The functional outcome at the discharge of those patients in whom multimodal monitoring was performed was favorable in a greater proportion than in those in whom it was not performed: 9/20 (45%) vs. 14/88 (16%) (*p* = 0.004).

### 3.6 Vasospasm, cerebral infarcts and DCI

Angiographic vasospasm was found in 34 patients (34/108; 31%) and was significantly associated with poor outcomes at discharge (*p* = 0.016) and at 3 months (*p* = 0.002). Furthermore, this was present more frequently in patients who suffered DCI (15/21; 71%) (*p* < 0.001), but not in those who had a cerebral infarct (20/52; 38%) (*p* = 0.132).

Cerebral infarcts were identified in 52 individuals (52/108; 48%). There was no statistically significant association when examining functional outcomes categorized as good (mRS 0–3) versus poor (mRS 4–6), both at discharge (*p* = 0.230) and at three-month follow-up (*p* = 0.084). DCI was diagnosed in 24 patients (22%). Similarly, no significant differences were noted in functional prognosis at discharge (*p* = 0.615) or after 3 months (*p* = 0.545). However, DCI showed a strong correlation with the presence of new cerebral infarcts, which occurred in 21 out of the 24 patients with DCI (90%; *p* < 0.001).

## 4 Discussion

In our cohort of poor grade aSAH (WFN 4–5), around 80% of patients had poor neurological outcomes at hospital discharge, and this was associated with older age, lower level of consciousness at admission and the development of DCI. In addition, 59% of patients aged 75 and older had limitations on therapeutic efforts, leading to a 100% mortality rate in that subgroup. In our cohort, patients who achieved a favorable outcome had an average age of 55 years, while those with a poor outcome had an average age of 61 years. Notably, patients undergoing multimodal monitoring had better functional outcomes at discharge.

### 4.1 Demographic and clinical characteristics

The percentage of patients admitted to hospitals with severe aSAH (WFNS 4–5) is approximately 20–30% of all aSAH cases, with a higher prevalence in women (around 60%). Smoking and cardiovascular risk factors, such as high blood pressure, are common among patients with this condition. These factors have been shown to be associated with a higher risk of developing cerebral aneurysms, as well as an increased risk of aneurysm rupture. However, it remains unclear whether they worsen the prognosis of patients whose clinical condition is initially poor after bleeding (22–24).

In our cohort, the distribution of aneurysm locations was similar to that described in the literature, with a predominance of ACom and MCA, followed by PCom aneurysms and, finally, those located in the vertebrobasilar system (2).

The overall mortality rate for patients with WFNS 4–5 in our cohort was approximately 50% following hospital admission, also consistent with findings from other studies (25, 26).

### 4.2 Age and limitation of therapeutic effort

In our cohort, younger age was shown to be a factor associated with prognosis. Conversely, when we analyzed age in relation to treated and untreated patients, this difference disappeared.

Interestingly, when we analyzed the prognostic effect of age in treated and untreated patients separately, this difference disappeared. This may be due to the role that age plays in critical patients regarding the decision to perform invasive procedures (such as clipping or embolization). As we observed, LTE was significantly more common among older individuals. In our study, LTE was implemented in 50% of patients over the age of 75, compared to only 8% in those younger than 75. This finding aligns with previous research by Lillemoe et al., who also noted a correlation between LTE and older age (27). In this line, Zahuarenc et al. indicated that LTE is an independent factor affecting mortality after a cerebral hemorrhage (28).

Moreover, no other disease-related factor seemed to influence the decision to implement LTE to such an extent as age; neither the location of the aneurysm nor did the presence of an intraparenchymal hematoma appeared to be a determining factors.

In our study, 17 out of 52 patients (32.7%) received treatment with LTE. Notably, this indicates that nearly one-third of the patients who died did not receive any therapeutic intervention. When comparing outcomes between treated and untreated patients, we observed a mortality rate of 77.3% (17 out of 22) among those who did not receive LET, while there were no deaths attributed to LTE among the patients who had been treated. This disparity may reflect a prevailing therapeutic nihilism in the management of patients presenting in such critical clinical conditions, particularly among older individuals. We consider it likely that more aggressive management from the outset, avoiding LTE, although everything indicates that we will not be able to change the patient’s prognosis, will increase the number of non-fatal cases, as well as the percentage of patients with a good functional prognosis.

### 4.3 Initial clinical status and functional prognosis

Patients with poor initial clinical status following aSAHs (WFNS grades 4–5) have significantly high morbidity and mortality rates, with estimates that up to 50% may not survive by the time they reach healthcare facilities (25, 26). Yet the percentage of these patients who survive with a good functional outcome has been increasing in recent years, reaching up to 30% (13, 16).

In our cohort, 20% of patients with poor clinical status at hospital admission had a good functional prognosis at discharge (23/108) and 32% at 3 months follow-up (35/108). According to several authors, early aneurysm treatment and neurocritical management of these patients could improve the functional outcome in a limited subgroup of patients with poor initial clinical conditions (17, 25, 29, 30). Despite that, it is difficult to initially select those patients who will have a good clinical evolution.

Regarding the initial WFNS score, there seems to be a tendency for patients with WFNS 4 to have a better functional prognosis at 3 months than those who present with WFNS 5. This former group might benefit more significantly from intensive measures. In our analysis the difference is not significant; however, Rabee et al. (31) propose subclassifying patients with WFNS 5 based on whether or not there is brain stem involvement, to improve the specificity.

Hydrocephalus as an initial clinical condition is an important factor in the prognosis of aSAH (32). The fact that we do not find this correlation in patients with an initial WFNS grade of 4 or 5 is probably due to the fact that the poor prognosis is already determined by the severity of the initial damage.

Both hydrocephalus and re-bleeding are initial clinical factors that influence the prognosis of subarachnoid hemorrhage (SAH); in our study, we did not find differences correlating re-bleeding with poor functional outcomes in this cohort of patients with poor initial grade. These results could be explained by the insufficient number of patients to identify significant differences.

### 4.4 Aneurysm treatment

Our results suggest that the aneurysm exclusion treatment improves the prognosis of patients with severe aSAH, regardless of the type of treatment. In our study, all patients who did not receive exclusion treatment ultimately died. It is important to acknowledge that this finding may be influenced by patient selection bias; specifically, those chosen for treatment tended to have a more favorable initial clinical profile regarding factors such as age and comorbidities.

The type of aneurysm treatment does not seem to significantly influence the clinical evolution of patients with aSAH. Although endovascular treatment could increase the risk of rebleeding, it is not very relevant in most studies (33–35). When we make this comparison in patients in poor initial clinical condition, this trend is maintained in both groups.

It has been observed that there is improved management of intracranial pressure and consequently a better prognosis following the performance of a craniotomy for hematoma evacuation and aneurysm clipping (36, 37). The presence of hematoma in our patients showed a trend toward poorer functional outcomes at discharge (*p* = 0.057); however, this trend did not persist at the three-month follow-up.

### 4.5 Multimodal monitoring

The use of multimodal monitoring is not a widespread practice in aSAH, probably due to the difficulties involved in its systematic placement and the correct interpretation of the different techniques, requiring close and coordinated collaboration between neurosurgery, neurology, anesthesia, intensive care unit and nursing services. In our experience, it has proven to be a useful and safe tool when managing these patients in the intensive care unit (17). In the case of a neurocritical patient requiring intensive and continuous treatment, having information that allows early detection of complications and their optimization, such as DCI, late infarctions or intracranial hypertension, is crucial and can make a significant difference (7, 13, 15, 38). In our study, we observed that patients undergoing multimodal monitoring had a better functional prognosis at discharge (*p* = 0.002) and at 3 months (*p* = 0.038). Although this is true, there could be a selection bias, since the placement of the monitoring was not immediate, so no patient with a poor prognosis in the first 24 h was selected for it. In addition, the fact of having the monitoring implanted favors more exhaustive clinical attention, which, added to the fact of having very precise information about the cerebral metabolic state almost in real time, could result in this prognostic improvement.

### 4.6 Vasospasm, cerebral infarction and DCI

The attention given to secondary brain injury (SBI) in the form of DCI or cerebral infarcts has increased in recent years. The appearance of DCI in a rather high percentage of aSAH patients and its prognostic implications highlight the need for early identification to offer immediate and optimized treatment that prevents irreparable ischemic damage or minimizes its consequences (39–41).

One of the objectives of our study was to describe how these phenomena behave in a patient who is admitted to a poor initial clinical condition. We correlated the appearance of infarcts with the increase in mRS 1–6 (*p* = 0.026); as well as with the presence of DCI. However, we did not find a direct correlation between the presence of infarcts and poor functional outcomes (mRS > 3). This may be attributed to the fact that the initial damage caused by bleeding in patients with WFNS 4–5 is significant enough to have a major impact on prognosis. Additionally, we observed a high incidence of new infarcts (48%), many in non-eloquent brain areas.

The lack of correlation between DCI and functional prognosis can be justified by the limitations of the definition of DCI itself. To determine DCI, it is essential to demonstrate neurological deterioration. However, for many patients who are sedated and intubated, this deterioration cannot be assessed effectively.

We observed a statistically significant correlation concerning the occurrence of aVE, as assessed through angiography, CT angiography, or transcranial Doppler ultrasound. It is important to note that these diagnostic modalities are not solely employed based on the deterioration of the patient’s neurological condition but also in response to various clinical changes or intensive monitoring requirements. Consequently, it is likely that a comprehensive neuromonitoring strategy, aimed at detecting aVE, may provide enhanced management insights and prognostic information compared to the mere observation of delayed ischemia in patients presenting with an initial poor clinical condition.

Follow-up data were collected up to 3 months after discharge, with better functional results at 3 months. In recent years, several studies have shown that patients with aSAH with a poor initial clinical condition can improve up to 1 year after hospital discharge (16, 42, 43).

## 5 Conclusion

The prognosis of patients with aSAH and poor clinical condition on admission is generally unfavorable. Invasive monitoring and tailored treatment appear to be beneficial in achieving favorable results in these patients. Despite the initial severity, up to 20% of patients achieve a good functional result on discharge and up to 35% do so at 3 months. These should be considered in the initial prognostic assessment with the families of these patients.

### 5.1 Limitations

This study presents several limitations that should be considered when interpreting its findings.

A primary limitation is the severity of the patients included in the study, which complicates the establishment of clinical correlations with the various variables examined.

From a methodological perspective, notable variations occurred in the management of aneurysm treatment throughout the study period. In the early years, endovascular approaches were predominantly used; however, there was a marked increase in the frequency of surgical interventions during the latter half of the study. This shift may reflect both changes in the treating team’s composition and the evolution of medical criteria over time.

Lastly, the sample size of patients receiving comprehensive multimodal monitoring is relatively small, highlighting the need for larger studies to obtain more robust data.

## Data Availability

The original contributions presented in the study are included in the article/supplementary material, further inquiries can be directed to the corresponding author.

## References

[1] Hutchinson PJ Seeley HM Kirkpatrick PJ. Factors implicated in deaths from subarachnoid haemorrhage: are they avoidable? Br J Neurosurg. (1998) 12:37–40. doi: 10.1080/02688699845492, PMID: 11013646

[2] Wiebers DO Whisnant JP Huston J 3rd Meissner I Brown RD Jr Piepgras DG. Unruptured intracranial aneurysms: natural history, clinical outcome, and risks of surgical and endovascular treatment. Lancet. (2003) 362:103–10. doi: 10.1016/S0140-6736(03)13860-3, PMID: 12867109

[3] Lu J Wang L Li R Lin F Chen Y Yan D. Timing of operation for poor-grade aneurysmal subarachnoid hemorrhage: relationship with delayed cerebral ischemia and poor prognosis. CNS Neurosci Ther. (2023) 29:1120–8. doi: 10.1111/cns.14088, PMID: 36627811 PMC10018093

[4] Rinkel GJE Algra A. Long-term outcomes of patients with aneurysmal subarachnoid haemorrhage. Lancet Neurol. (2011) 10:349–56. doi: 10.1016/S1474-4422(11)70017-5, PMID: 21435599

[5] Steiner T Juvela S Unterberg A Jung C Forsting M Rinkel G. European stroke organization guidelines for the management of intracranial aneurysms and subarachnoid haemorrhage. Cerebrovasc Dis. (2013) 35:93–112. doi: 10.1159/000346087 23406828

[6] Ropper AH Zervas NT. Outcome 1 year after SAH from cerebral aneurysm. Management morbidity, mortality, and functional status in 112 consecutive good-risk patients. J Neurosurg. (1984) 60:909–15. doi: 10.3171/jns.1984.60.5.0909, PMID: 6716158

[7] Francoeur CL Mayer SA. Management of delayed cerebral ischemia after subarachnoid hemorrhage. Crit Care. (2016) 20:277. doi: 10.1186/s13054-016-1447-6, PMID: 27737684 PMC5064957

[8] Ahn SH Savarraj JP Pervez M Jones W Park J Jeon SB. The subarachnoid hemorrhage early brain edema score predicts delayed cerebral ischemia and clinical outcomes. Neurosurgery. (2018) 83:137–45. doi: 10.1093/neuros/nyx364, PMID: 28973675

[9] de Oliveira Manoel AL Jaja BN Germans MR Yan H Qian W Kouzmina E. The VASOGRADE: a simple grading scale for prediction of delayed cerebral ischemia after subarachnoid hemorrhage. Stroke. (2015) 46:1826–31. doi: 10.1161/STROKEAHA.115.008728, PMID: 25977276

[10] Zhou J Guo P Guo Z Sun X Chen Y Feng H. Fluid metabolic pathways after subarachnoid hemorrhage. J Neurochem. (2022) 160:13–33. doi: 10.1111/jnc.15458, PMID: 34160835

[11] Zhou T Kalanuria A. Cerebral microdialysis in Neurocritical care. Curr Neurol Neurosci Rep. (2018) 18:101. doi: 10.1007/s11910-018-0915-6, PMID: 30353361

[12] Kramer AH Couillard PL Kromm JA Ruddell S Demers-Marcil S Mitha AP. Findings predictive of poor outcome in grade 5 subarachnoid hemorrhage: a cohort study. Can J Neurol Sci. (2021):1–10. doi: 10.1017/cjn.2021.13, PMID: 33472716

[13] Rass V Helbok R. Early brain injury after poor-grade subarachnoid hemorrhage. Curr Neurol Neurosci Rep. (2019) 19:78. doi: 10.1007/s11910-019-0990-3, PMID: 31468197 PMC6715808

[14] Zheng K Zhong M Zhao B Chen SY Tan XX Li ZQ. Poor-grade aneurysmal subarachnoid hemorrhage: risk factors affecting clinical outcomes in intracranial aneurysm patients in a multi-center study. Front Neurol. (2019) 10:123. doi: 10.3389/fneur.2019.00123, PMID: 30873104 PMC6400833

[15] Schuss P Hadjiathanasiou A Borger V Wispel C Vatter H Güresir E. Poor-grade aneurysmal subarachnoid hemorrhage: factors influencing functional outcome—a single-center series. World Neurosurg. (2016) 85:125–9. doi: 10.1016/j.wneu.2015.08.046, PMID: 26341439

[16] de Oliveira Manoel AL Mansur A Silva GS Germans MR Jaja BNR Kouzmina E. Functional outcome after poor-grade subarachnoid hemorrhage: a single-center study and systematic literature review. Neurocrit Care. (2016) 25:338–50. doi: 10.1007/s12028-016-0305-3, PMID: 27651379

[17] Torné R Culebras D Sanchez-Etayo G García-García S Muñoz G Llull L. Double hemispheric microdialysis study in poor-grade SAH patients. Sci Rep. (2020) 10:7466. doi: 10.1038/s41598-020-64543-x, PMID: 32366972 PMC7198586

[18] Torné R Hoyos J Llull L Rodríguez-Hernández A Muñoz G Mellado-Artigas R. Edema resolution and clinical assessment in poor-grade subarachnoid hemorrhage: useful indicators to predict delayed cerebral infarctions? J Clin Med. (2021) 10:321. doi: 10.3390/jcm10020321 33477258 PMC7830766

[19] Hutchinson PJ Jalloh I Helmy A Carpenter KLH Rostami E Bellander BM. Consensus statement from the 2014 international microdialysis forum. Intensive Care Med. (2015) 41:1517–28. doi: 10.1007/s00134-015-3930-y, PMID: 26194024 PMC4550654

[20] Rass V Solari D Ianosi B Gaasch M Kofler M Schiefecker AJ. Protocolized brain oxygen optimization in subarachnoid hemorrhage. Neurocrit Care. (2019) 31:263–72. doi: 10.1007/s12028-019-00753-0, PMID: 31218640 PMC6757026

[21] García-García S Culebras D Torné R. Letter to the editor. Invasive neuromonitoring for poor-grade SAH. J Neurosurg. (2021) 134:1679–80. doi: 10.3171/2020.5.JNS202047, PMID: 32764169

[22] Hoh BL Ko NU Amin-Hanjani S Chou SHY Cruz-Flores S Dangayach NS. 2023 guideline for the Management of Patients with Aneurysmal Subarachnoid Hemorrhage: a guideline from the American Heart Association/American Stroke Association. Stroke. (2023) 54:e314–70. doi: 10.1161/STR.0000000000000436, PMID: 37212182

[23] Osgood ML. Aneurysmal subarachnoid hemorrhage: review of the pathophysiology and management strategies. Curr Neurol Neurosci Rep. (2021) 21:50. doi: 10.1007/s11910-021-01136-9, PMID: 34308493

[24] Karhunen V Bakker MK Ruigrok YM Gill D Larsson SC. Modifiable risk factors for intracranial aneurysm and aneurysmal subarachnoid hemorrhage: a Mendelian randomization study. J Am Heart Assoc. (2021) 10:e022277. doi: 10.1161/JAHA.121.022277, PMID: 34729997 PMC8751955

[25] Hoogmoed J Coert BA van den Berg R Roos YBWEM Horn J Vandertop WP. Early treatment decisions in poor-grade patients with subarachnoid hemorrhage. World Neurosurg. (2018) 119:e568–73. doi: 10.1016/j.wneu.2018.07.212, PMID: 30077026

[26] Lantigua H Ortega-Gutierrez S Schmidt JM Lee K Badjatia N Agarwal S. Subarachnoid hemorrhage: who dies, and why? Crit Care. (2015) 19:309. doi: 10.1186/s13054-015-1036-0, PMID: 26330064 PMC4556224

[27] Lillemoe K Lord A Torres J Ishida K Czeisler B Lewis A. Factors associated with DNR status after nontraumatic intracranial hemorrhage. Neurohospitalist. (2020) 10:168–75. doi: 10.1177/1941874419873812, PMID: 32549939 PMC7271616

[28] Zahuranec DB Brown DL Lisabeth LD Gonzales NR Longwell PJ Smith MA. Early care limitations independently predict mortality after intracerebral hemorrhage. Neurology. (2007) 68:1651–7. doi: 10.1212/01.wnl.0000261906.93238.72, PMID: 17502545

[29] de Winkel J Cras TY Dammers R van Doormaal PJ van der Jagt M Dippel DWJ. Early predictors of functional outcome in poor-grade aneurysmal subarachnoid hemorrhage: a systematic review and meta-analysis. BMC Neurol. (2022) 22:239. doi: 10.1186/s12883-022-02734-x, PMID: 35773634 PMC9245240

[30] Al-Mufti F Mayer SA Kaur G Bassily D Li B Holstein ML. Neurocritical care management of poor-grade subarachnoid hemorrhage: unjustified nihilism to reasonable optimism. Neuroradiol J. (2021) 34:542–51. doi: 10.1177/19714009211024633, PMID: 34476991 PMC8649190

[31] Raabe A Beck J Goldberg J Graggen ZWJ Branca M Marbacher S. Herniation world Federation of Neurosurgical Societies Scale Improves Prediction of outcome in patients with poor-grade aneurysmal subarachnoid hemorrhage. Stroke. (2022) 53:2346–51. doi: 10.1161/STROKEAHA.121.036699, PMID: 35317612

[32] Germanwala AV Huang J Tamargo RJ. Hydrocephalus after aneurysmal subarachnoid hemorrhage. Neurosurg Clin N Am. (2010) 21:263–70. doi: 10.1016/j.nec.2009.10.013, PMID: 20380968

[33] Souza S. Aneurysmal subarachnoid hemorrhage. J Neurosurg Anesthesiol. (2015) 27:222–40. doi: 10.1097/ANA.0000000000000130 25272066 PMC4463029

[34] Molyneux A Kerr R Stratton I Sandercock P Clarke M Shrimpton J. International subarachnoid aneurysm trial (ISAT) of neurosurgical clipping versus endovascular coiling in 2143 patients with ruptured intracranial aneurysms: a randomised trial. Lancet. (2002) 360:1267–74. doi: 10.1016/S0140-6736(02)11314-6, PMID: 12414200

[35] van der Schaaf I Algra A Wermer M Molyneux A Clarke M van Gijn J. Endovascular coiling versus neurosurgical clipping for patients with aneurysmal subarachnoid haemorrhage. Cochrane Database Syst Rev. (2005) 4:CD003085. doi: 10.1002/14651858.CD003085.pub2, PMID: 16235314

[36] Masson RL Day AL. Aneurysmal intracerebral hemorrhage. Neurosurg Clin N Am. (1992) 3:539–50. doi: 10.1016/S1042-3680(18)30645-4, PMID: 1633478

[37] Darkwah Oppong M Skowronek V Pierscianek D Gembruch O Herten A Saban DV. Aneurysmal intracerebral hematoma: risk factors and surgical treatment decisions. Clin Neurol Neurosurg. (2018) 173:1–7. doi: 10.1016/j.clineuro.2018.07.014, PMID: 30053744

[38] Helbok R Madineni RC Schmidt MJ Kurtz P Fernandez L Ko SB. Intracerebral monitoring of silent infarcts after subarachnoid hemorrhage. Neurocrit Care. (2011) 14:162–7. doi: 10.1007/s12028-010-9472-9, PMID: 21125348

[39] Geraghty JR Testai FD. Delayed cerebral ischemia after subarachnoid hemorrhage: beyond vasospasm and towards a multifactorial pathophysiology. Curr Atheroscler Rep. (2017) 19:50. doi: 10.1007/s11883-017-0690-x, PMID: 29063300

[40] Chen HY Elmer J Zafar SF Ghanta M Moura Junior V Rosenthal ES. Combining transcranial Doppler and EEG data to predict delayed cerebral ischemia after subarachnoid hemorrhage. Neurology. (2022) 98:e459–69. doi: 10.1212/WNL.0000000000013126, PMID: 34845057 PMC8826465

[41] Chou SHY. Subarachnoid hemorrhage. Continuum. (2021) 27:1201–45. doi: 10.1212/CON.0000000000001052 34618758

[42] Gouvea Bogossian E Battaglini D Fratino S Minini A Gianni G Fiore M. The role of brain tissue oxygenation monitoring in the Management of Subarachnoid Hemorrhage: a scoping review. Neurocrit Care. (2023) 39:229–40. doi: 10.1007/s12028-023-01680-x, PMID: 36802011

[43] Wilson DA Nakaji P Albuquerque FC McDougall CG Zabramski JM Spetzler RF. Time course of recovery following poor-grade SAH: the incidence of delayed improvement and implications for SAH outcome study design. J Neurosurg. (2013) 119:606–12. doi: 10.3171/2013.4.JNS121287, PMID: 23724983

